# Differential Expression of Human Peripheral Mononuclear Cells Phenotype Markers in Type 2 Diabetic Patients and Type 2 Diabetic Patients on Metformin

**DOI:** 10.3389/fendo.2018.00537

**Published:** 2018-10-09

**Authors:** Mohammed S. Al Dubayee, Hind Alayed, Rana Almansour, Nora Alqaoud, Rahaf Alnamlah, Dana Obeid, Awad Alshahrani, Mahmoud M. Zahra, Amre Nasr, Ahmad Al-Bawab, Ahmad Aljada

**Affiliations:** ^1^College of Medicine, King Saud bin Abdulaziz University for Health Sciences, Riyadh, Saudi Arabia; ^2^King Abdullah Specialized Children Hospital, Ministry of National Guard Health Affairs, Riyadh, Saudi Arabia; ^3^King Abdullah International Medical Research Center, Riyadh, Saudi Arabia

**Keywords:** monocyte subtypes, metabolic syndrome, atherosclerosis, inflammation, metabolically-activated macrophages

## Abstract

**Background:** Although peripheral blood mononuclear cells (PBMC) have been demonstrated to be in a pro-inflammatory state in obesity and type 2 Diabetes Mellitus (T2DM), characterization of circulating PBMC phenotypes in the obese and T2DM and the effect of Metformin on these phenotypes in humans is still ill-defined and remains to be determined.

**Methods:** Thirty normal healthy adult volunteers of normal weight, 30 obese subjects, 20 obese newly diagnosed diabetics and 30 obese diabetics on Metformin were recruited for the study. Fasting blood samples were collected and PBMC were isolated from whole blood. Polarization markers (CD86, IL-6, TNFα, iNOS, CD36, CD11c, CD169, CD206, CD163, CD68, CD11b, CD16, and CD14) were measured by RT-qPCR. Gene expression fold changes were calculated using the 2^−ΔΔ*CT*^ method for RT-qPCR.

**Results:** Obesity and T2DM are associated an increased CD68 marker in PBMC. mRNA expression of CD11b, CD11c, CD169, and CD163 were significantly reduced in PBMC from T2DM subjects whereas CD11c was significantly inhibited in PBMC from obese subjects. On the other hand, macrophage M1-like phenotype was observed in T2DM circulation as demonstrated by increased mRNA expression of CD16, IL-6, iNOS, TNFα, and CD36. There were no significant changes in CD14 and CD86 in the obese and T2DM when compared to the lean subjects. Metformin treatment in T2DM reverted CD11c, CD169, IL-6, iNOS, TNFα, and CD36 to levels comparable to lean subjects. CD206 mRNA expression was significantly upregulated in PBMC of T2DM while Metformin treatment inhibited CD206 expression levels.

**Conclusions:** These data support the notion that PBMC in circulation in T2DM express different pattern of phenotypic markers than the patterns typically present in M1 and M2 like cells. These phenotypic markers could be representative of metabolically activated macrophages (MMe)-like cells. Metformin, on the other hand, reduces MMe-like cells in circulation.

## Introduction

PBMC in blood consist of a mixed population of white blood cells comprised by monocytes (20%) and lymphocytes (T and B cells) and other cells of lymphoid origins. Three subsets of human monocytes in circulation have been recently described, based on the expression of the surface markers CD14 and CD16 (1). The characterization and the specific roles for these subsets in homeostasis and inflammation is still ill-defined and in its infancy. The major population of human monocytes (80–90%) constitute the “classical monocytes” and express high levels of CD14 and are devoid of surface CD16 expression and are phagocytic with no inflammatory attributes (2). The other remaining 10–20% of human monocytes population have been further classified into two subtypes. The more abundant “non-classical monocytes” with relatively low expression of surface CD14 and high levels of CD16 and display inflammatory features (1, 2). The third monocyte subtype, called “intermediate monocytes,” express high levels of both surface CD14 and CD16 (2).

Inflammation plays a major role in atherosclerosis mediated by PBMC. While monocytes form the initial inflammatory lesion to the endothelium in the process of atherogenesis, T cells and possibly B cells also participate in this process (3). As atherosclerotic plaques increase in size, PBMC recruitment continues to be evident, predominantly at plaque borders (4). The localized nature and PBMC specificity of these cellular interactions may, in part, be a consequence of changes in the adhesive properties of the endothelial surface that involve the expression of inducible endothelial-leucocyte adhesion molecules that are expressed in atherosclerotic plaques. Recruitment of circulating monocyte into peripheral tissue, as a response to cytokines, promote their differentiation into mature tissue-specific macrophages (5). Mature tissue-specific macrophages are indicated by phenotypic heterogeneity and reveal a spectrum of activation programs that present as a function of their different micro-environmental stimuli, including fate-determining growth factors, pro-inflammatory cytokines, and microbial components (6). Naïve macrophages (M0) can polarize into “classically activated” pro-inflammatory macrophages (M1), “alternatively activated” anti-inflammatory macrophages (M2) and the newly described “metabolically activated” macrophage phenotype (MMe) in response to different environmental stimuli as demonstrated in human adipose macrophages (7).

The M1 phenotype, expressing unique surface markers such as CD80, and CD86, is activated by Th1-derived interferon-gamma (IFNγ) and lipopolysaccharides (8, 9). It secretes pro-inflammatory cytokines, such as tumor necrosis factor (TNFα), interleukin-1 (IL-1), and IL-6, that are implicated in initiating and sustaining inflammatory functions (10). On the other hand, the M2 are induced by exposure to IL-4 and IL-13 and exert anti-inflammatory effects. M2 macrophages exert strong IgE response and are involved in fungal and parasitic infections and tissue remodeling (11). Furthermore, M2 are largely characterized by inhibition of the major surface markers expression usually found in M1 phenotype (12). Interestingly, the two macrophage phenotypes metabolize arginine differently as M1 macrophages convert arginine to nitric oxide (NO) while M2 macrophages convert arginine to ornithine (13, 14). Excessive NO production damages lipids, proteins and DNA and inhibits cell division whereas ornithine production stimulates cell division and wound healing (15, 16).

MMe have been recently described as pro-inflammatory phenotype expressed in response to treatment with glucose, insulin, and palmitate (7). However, MMe exhibits characteristics present in M1 and M2 phenotypes. MMe does not express the classic markers of M1 macrophages although they produce large amounts of pro-inflammatory cytokines in response to metabolic conditions in human adipose macrophages (7). On the other hand, MMe cell surface markers expression is regulated by PPAR-γ and p62/SQSTM1, which are known to exert an inhibitory effect and are associated with the alternative activation of M2 in mice (7, 17).

Obesity and T2DM have similar metabolic milieu hallmarked by insulin resistance (18, 19). Obesity and T2DM are associated with a low chronic subclinical inflammatory state, which is mediated by inflammatory mediators (e.g., TNFα, IL-6, iNOS, and C-reactive protein) produced by adipose tissue (20–23), macrophage infiltration, and insulin insensitivity in adipose tissue (18). Moreover, overnutrition causes expansion of adipocytes which secrete pro-inflammatory mediators (e.g., TNFα and IL-6) and chemokines [e.g., monocyte chemoattractant protein-1 (MCP-1)], thus increasing the recruitment of monocytes into adipose tissue (19). In human adipose tissue, macrophages have been shown to be of an anti-inflammatory phenotype (M2) but these M2 macrophages possess a strong capacity to produce pro-inflammatory mediators (24). In mice, the M1:M2 ratio is 1:4 in normal lean mice whereas it progressively increases to 1.2:1 in obesity/diabetes (25). Weight loss, on the other hand, leads to the reversal of inflammation in human (26).

Metformin (1,1-dimethylbiguanide) is the most commonly used anti-diabetic drug for the treatment of T2DM. Metformin has been shown to have anti-inflammatory effects (27–29). Furthermore, Metformin significantly reduces the risk of cardiovascular complications that are related to T2DM (30). In this study, we hypothesized that obesity and the metabolic syndrome modulate PBMC phenotypic characteristics resulting from metabolic activation. In addition, we hypothesized that Metformin would alter PBMC phenotype markers in circulation as it exerts anti-inflammatory properties in T2DM.

## Materials and methods

### Subjects

Thirty lean adult volunteers of normal weight, 30 obese adult volunteers with normal glucose levels and with no medications, 20 drug-naïve obese T2DM newly diagnosed volunteers, 30 obese T2DM on Metformin volunteers were recruited for the study. The majority of T2DM on Metformin were on Metformin for at least 9 months and up to 15 years. Most of subjects on Metformin regiments comprised of dosages which varied from 1,000 to 2,000 mg, five had dosages ranging from 100 to 500 daily, and three had dosages ranging from 3,000 to 4,500 mg daily. Five T2DM on Metformin subjects were on insulin therapy as well. The study was approved by the IRB of King Abdulaziz Medical City. Subjects gave their written informed consent.

### Isolation of PBMC

Ten mL of the anti-coagulated blood sample (Na-EDTA) were diluted with an equal volume of Phosphate Buffered Saline (PBS) and were carefully layered over 15 mL of Ficol-Hypaque (50 mL Leucosep Tubes, Greiner Bio-One North America Inc, North Carolina, USA). Samples were centrifuged at 450 × g, in a swing out rotor for 30 min at 22°C and the PBMC layer was harvested with a pipette. PBMC were repeatedly washed with PBS. Fifty μL of Qiagen RNALater were added to the pellet and samples were then frozen at −80°C.

### Quantitative real time polymerase chain reaction (qRT-PCR) analysis

Total RNA was isolated using the Ambion Aqueous kit (Ambion). All isolated RNA samples were treated with DNase I to remove contaminating genomic DNA. The quality and quantity of the isolated RNA was determined using Agilent Bioanalyzer 2100. One μg of total RNA was reverse-transcribed using first strand cDNA synthesis Kit (Millipore, USA) followed by real time quantitative PCR (qRT-PCR). qRT-PCR was performed with a 7900HT Fast Real-Time PCR System (Applied Biosystems, USA), using 2 μL cDNA, 10 μL 2X Sybergreen Master mix (150 mM Tris, pH 9.2, 40 mM (NH_4_)_2_SO_4_, 5 mM MgCl_2_, 0.02% Tween-20, 0.4 mM dNTPs, 1.25 Units Taq Polymerase, 1X Sybergreen) and 0.5 μL of 20 μM gene-specific primers (Table 1). Primers were designed based on theoretical optimal conditions, which included primer melting temperature, primer annealing temperature, GC content, cross homology and primer secondary structures. All primers were purchased from Bio-Basic Canada Inc. (Ontario, Canada). The specificity and size of the PCR products were tested by adding a melt curve at the end of the amplifications, analysis on a 2% agarose gel of the bands. Amplicon Bands were isolated and sequenced. The reaction protocol consisted of one activation cycle of 50°C for 2 min followed by 95°C for 15 s. Thereafter, 40 cycles of denaturation at 95°C for 15 s, and at 60°C annealing/extension for 2 min were performed. Although normalization to RPL13 and Ubiquitin C showed similar trends, all values were normalized to Ubiquitin C. The 2^−ΔΔ*CT*^ method was used for relative quantification for qRT-PCR experiments (31).

**Table 1:** Primer sequences for all primers used in RT-qPCR.

**Primer**	**Sense (5^′^ → 3^′^)**	**Anti sense (5^′^ → 3^′^)**	**Accession number**
CD68	GCTACATGGCGGTGGAGTACAA	ATGATGAGAGGCAGCAAGATGG	NM_001251.2
CD11b	CAGCCTTTGACCTTATGTCATGG	CCTGTGCTGTAGTCGCACT	NM_001145808.1
CD11c	CGTTCGACACATCCGTGTA	TTTGCCTCCTCCATCATTTC	NM_001286375.1
CD169	CCTCGGGGAGGAACATCCTT	AGGCGTACCCCATCCTTGA	NM_023068.3
CD163	CAGGAAACCAGTCCCAAACA	AGCGACCTCCTCCATTTACC	NM_004244.5
CD206	TTCGGACACCCATCGGAATTT	CACAAGCGCTGCGTGGAT	NM_002438.3
CD14	AGCCAAGGCAGTTTGAGTCC	TAAAGGACTGCCAGCCAAGC	NM_000591.3
CD16	ATGTGTCTTCAGAGACTGTGAAC	TTTATGGTCCTTCCAGTCTCTTG	NM_000569.7
CD86	CTGCTCATCTATACACGGTTACC	GGAAACGTCGTACAGTTCTGTG	NM_175862.4
IL-6	AATAACCACCCCTGACCCAAC	AATCTGAGGTGCCCATGCTAC	NM_000600.4
iNOS	TCCGAGGCAAACAGCACATTCA	GGGTTGGGGGTGTGGTGATGT	NM_000625.4
TNFα	CCTGCCCCAATCCCTTTATT	CCCTAAGCCCCCAATTCTCT	NM_000594.3
CD36	GCCAAGGAAAATGTAACCCAGG	GCCTCTGTTCCAACTGATAGTGA	NM_001001548.2
Ubiquitin	ACTACAACATCCAGAAAGAGTCCA	CCAGTCAGGGTCTTCACGAAG	NM_021009.6
RPL13	AACAAGTTGAAGTACCTGGCTTTC	TGGTTTTGTGGGGCAGCATA	NM_000977.3

### Statistical analysis

Statistical analysis was carried out using SigmaStat software ver. 3.0 (Jandel Scientific, San Rafael, CA). Fold change in mRNA expression was calculated for qRT-PCR results and analysis was carried out using One Way ANOVA followed by (Holm-Sidak method) for pairwise comparisons and comparison against the lean group. When normality distribution failed, One Way ANOVA on Ranks was run followed by Dunn's test for pairwise comparisons and comparison against the lean group. *P*-value < 0.05 was used to assess significance for all statistical analyses. Results are presented as mean ± S.E.M.

## Results

### Demographic data of subjects

Although there were significant differences in the age (Table 2), there were no significant correlation between the expression levels of any phenotypic markers examined in the study and age. Since five T2DM on Metformin subjects were on insulin therapy as well, exclusion of these subjects from the analysis resulted in similar results and thus were included in the analysis.

**Table 2:** Demographic data of the patients participated in the study.

	**Gender**	**Age**	**BMI**	**Glucose**	**LDL**	**HDL**	**Triglycerides**	**Hb_A1c_**	**Insulin**
		**(years)**	**kg/m^2^**	**(mmol/L)**	**(mmol/L)**	**(mmol/L)**	**(mmol/L)**	**(%)**	**(μU/mL)**
Lean	18M, 12F	25.7 ± 1.1	23.0 ± 0.3	5.1 ± 0.1	2.54 ± 0.26	1.35 ± 0.04	0.83 ± 0.09	5.8 ± 0.10	4.8 ± 0.60
Obese	11M, 19F	35.1 ± 2.3	39.1 ± 1.7*	5.4 ± 0.1	3.04 ± 0.16	1.18 ± 0.05	1.22 ± 0.12	5.6 ± 0.25	10.0 ± 1.5*
T2DM	15M, 5F	48.4 ± 3.0*^σ^	32.5 ± 1.9*	10.0 ± 1.1*^σ^	3.53 ± 0.19*	1.02 ± 0.05*^σ^	1.97 ± 0.23*^σ^	8.0 ± 0.62*^σ^	7.5 ± 2.6*
T2DM + Metformin	12M, 18F	47.1 ± 2.0*^σ^	40.5 ± 1.5*	10.0 ± 0.8*^σ^	2.60 ± 0.17^δ^	0.99 ± 0.04*^σ^	1.56 ± 0.16*	8.7 ± 0.35*^σ^	7.8 ± 1.5*

Results are presented as Mean ± S.E.M.

* *P < 0.05 vs. lean subjects*;

σ *P < 0.05 vs. obese*;

δ *P < 0.05 vs. T2DM*.

### mRNA expression of phenotype markers

**Figure 1:**
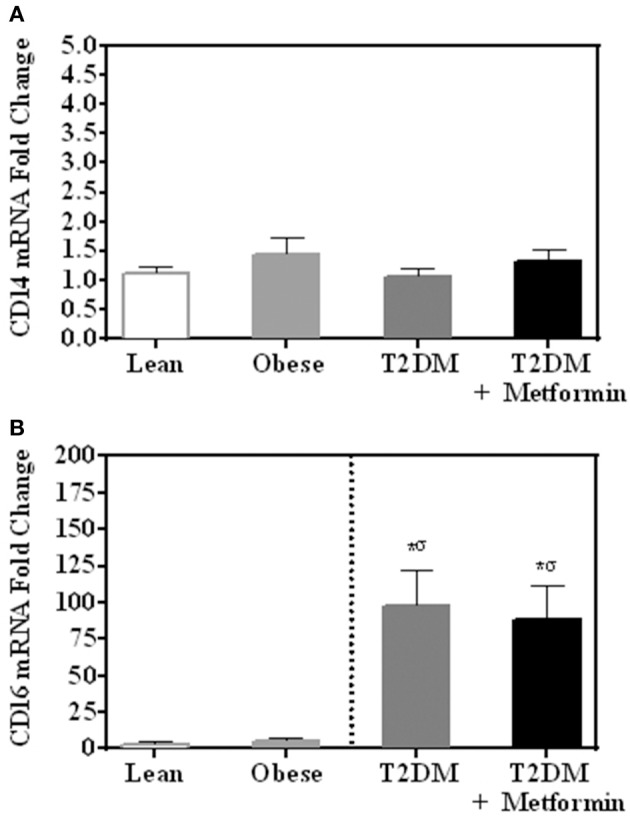
mRNA expression of **(A)** CD14 and **(B)** CD16 in PBMC of lean, obese, T2DM and T2DM on Metformin. Circulating PBMC in T2DM are associated with increased levels of CD16 mRNA expression. Metformin treatment did not reduce CD16 expression in PBMC significantly. Results are presented as mean ± S.E.M. *P<*0.05* vs. lean subjects; ^σ^*P*<*0.05* vs. obese.

There were no differences in mRNA expression of CD14 among lean, obese, T2DM, and T2DM on Metformin. CD16 expression (357 ± 94-fold change) was significantly higher in PBMC of T2DM (*P*<*0.001*) when compared to normal lean subjects and Metformin treatment did not reduce CD16 expression in T2DM significantly (Figures 1A,B). Since CD14 expression is normally high in monocytes of lean subjects as 80–90% of circulating monocytes are “classical monocyte” subtypes and the four groups had comparable expression levels, it is safe to conclude that CD14 expression is also high in the obese and T2DM.

**Figure 2:**
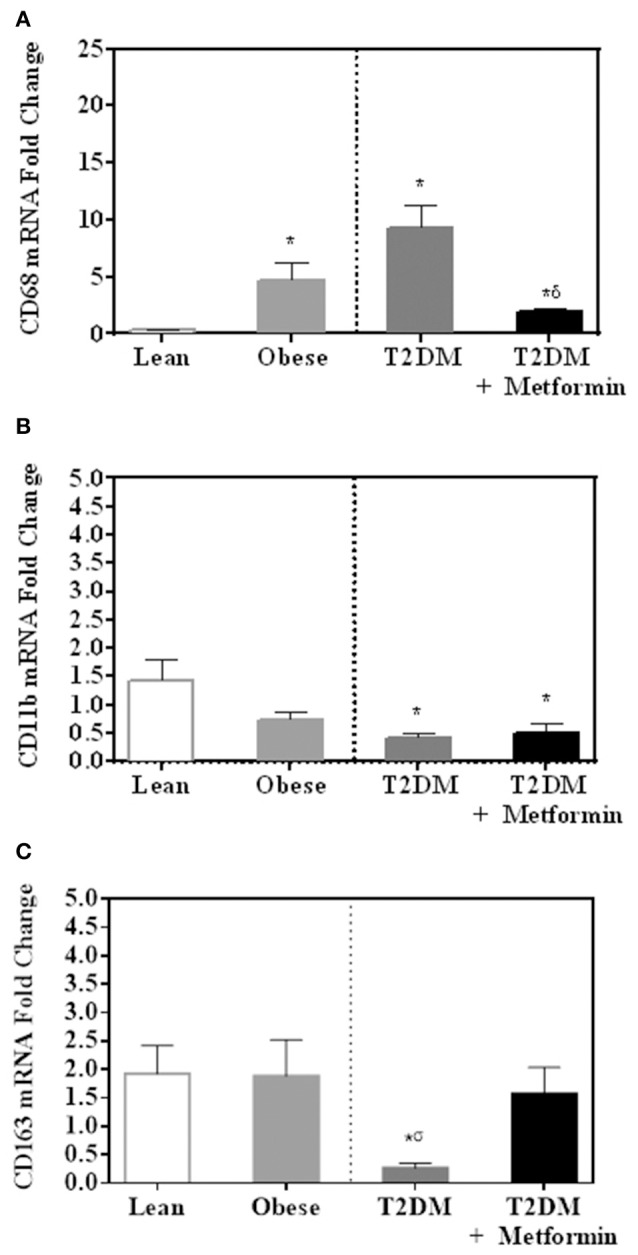
mRNA expression of several pan-macrophage markers in PBMC of lean, obese, T2DM and T2DM on Metformin [**(A)** CD68; **(B)** CD11b; and **(C)** CD163]. Metformin treatment inhibited CD68 mRNA expression significantly and did not change CD11b nor CD163 mRNA expression. Results are presented as mean ± S.E.M. *P<*0.05* vs. lean subjects; ^σ^*P*<*0.05* vs. obese; ^δ^*P*<*0.05* vs. T2DM.

Several pan-macrophage markers, CD68, CD11b, and CD163, were measured in PBMC of obese and obese T2DM and compared to PBMC of lean subjects. Obesity and T2DM were associated with increased CD68 marker expression (*P*<*0.001*, Figure 2A) while CD11b and CD163 expression were significantly inhibited in T2DM (*P*<*0.005*, Figures 2B,C). Metformin treatment, on the other hand, changed PBMC phenotypes in circulation as indicated by lower levels of CD68 and CD163 when compared to T2DM (Figures 2A,C) while it did not change CD11b expression significantly (Figure 2B).

**Figure 3:**
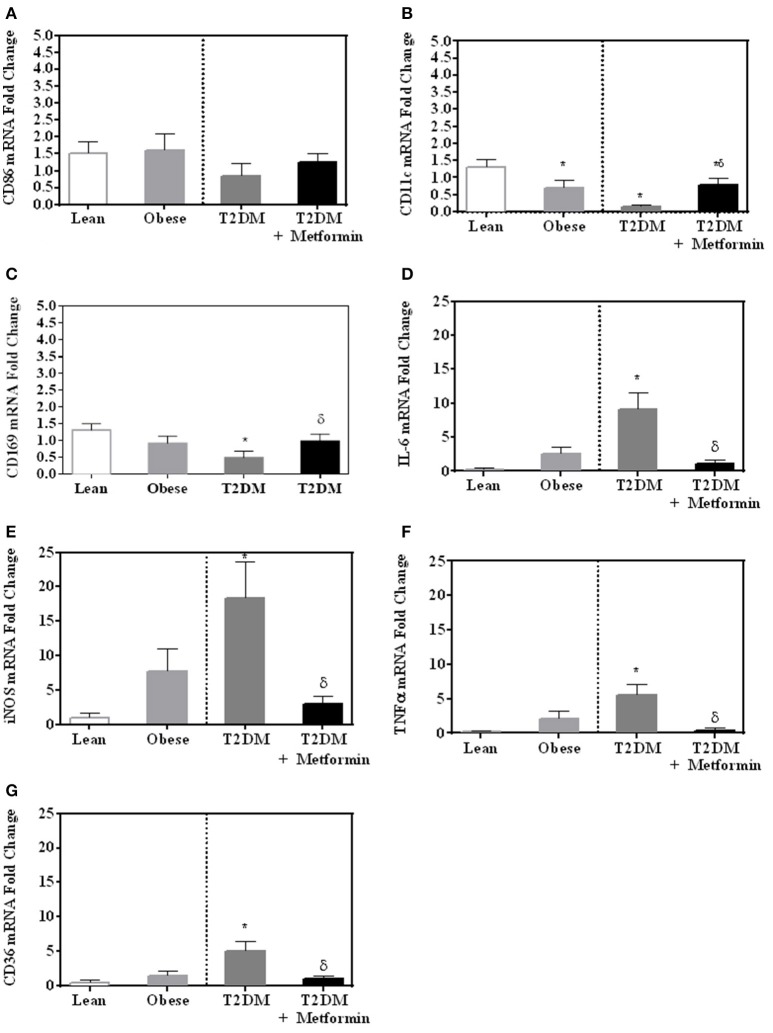
M1-like phenotype was evaluated in obesity and T2DM by measuring mRNA expression of **(A)** CD86; **(B)** CD11c; **(C)** CD169; **(D)** IL-6, **(E)** iNOS, **(F)** TNFα, and **(G)** CD36. Results are presented as mean ± S.E.M. *P < 0.05 vs. lean subjects; ^δ^*P* < 0.05 vs. T2DM.

**Figure 4:**
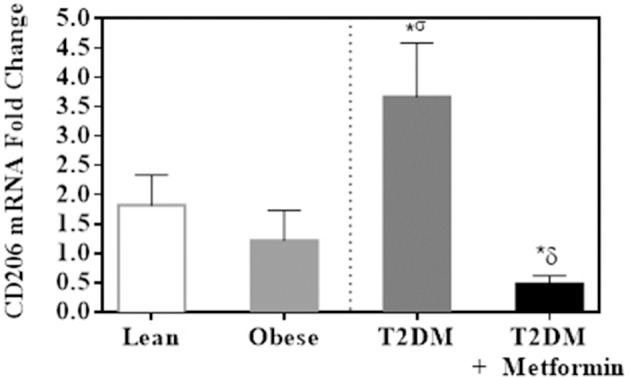
M2 phenotype marker, CD206, was significantly upregulated in PBMC of T2DM. Results are presented as mean ± S.E.M. *P < 0.05 vs. lean subjects; ^σ^*P* < 0.05 vs. obese; ^δ^*P* < 0.05 vs. T2DM.

Since both obesity and T2DM are pro-inflammatory states, we examined the expression of several M1 phenotype markers in PBMC of lean, obese, T2DM, and T2DM on Metformin, including CD86, CD11c, CD169, IL-6, TNFα, iNOS, and CD36. There were no significant changes in CD86 expression in obesity and T2DM when compared to PBMC of lean subjects (Figure 3A). On the other hand, CD11c expression was inhibited significantly in the obese and T2DM while CD169 expression was inhibited significantly only in PBMC of T2DM (*P*<*0.001*, Figures 3B,C). Circulating PBMC in T2DM had M1-like phenotype as demonstrated by increased expression of IL-6, iNOS, TNFα, and CD36 (thrombospondin receptor; *P*<*0.05*, Figures 3D–G). Metformin treatment reduced CD11c and CD169 inhibition in PBMC of T2DM (*P*<*0.05*, Figures 3B,C) and reduced the increased expression of IL-6, iNOS, TNFα, and CD36 (Figures 3D–G). The M2 phenotype marker examined in this study, CD206, was significantly upregulated in T2DM and Metformin treatment was associated with lower levels of CD206 (Figure 4). These data support the notion that metabolically activated MMe-like PBMC phenotype in T2DM are associated with increased levels of CD68, IL-6, iNOS, TNFα, CD206, and CD36 and decreased levels of CD11b, CD163, CD11c, CD169. These metabolically activated MMe-like PBMC are most likely containing “intermediate monocytes” since they have high expression of CD14 and CD16. Metformin treatment inhibited MMe-like phenotype as indicated by reversal of mRNA inhibition of CD11c and CD169 and reduction in increased expression of CD68, IL-6, iNOS, TNFα, CD206 and CD36.

## Discussion

Obesity and T2DM have been shown to be associated with an increase in pro-inflammatory cytokines and transcription factors in PBMC, e.g., NFκB. To assess further the presence of inflammation at the cellular and molecular level, we have now investigated the expression of many CD markers and cytokines by PBMC in an attempt to characterize their phenotypes. Contradictions with respect to white blood cells subset phenotypes and function have been reported. These contradictions stem from discrepancies in reliable gating strategies for flow cytometric characterization, antibodies specificities, and purification protocols resulting in changes in receptor expression. For example, Ficoll purification of blood monocytes could contribute to these contradictions as it could decrease CD14+/CD16– “classical monocytes” with a concomitant expansion of CD14dim/CD16+ “non-classical monocytes” (2). Our results demonstrated lack of differences in CD14 expression among the four groups tested while CD16 expression was induced significantly in PBMC of T2DM. CD14 is widely used as a monocyte/macrophage marker (32). Since CD14 expression is high in the lean subjects as “classical monocytes” constitute 80–90% of all circulating monocytes and CD14 expression is comparable in the four groups studied, we could conclude that CD14 expression is also high in PBMC of T2DM. This suggests an increase in the “intermediate monocytes” subtype as both CD14 and CD16 expression are high. However, since CD16 is expressed in NK, T-cell types and monocytes, cells other than monocytes could have increased levels of expression of CD16. Further studies are needed to support the notion that “intermediate monocytes” could get metabolically activated. This heterogeneity of cells utilized in this study represents a significant shortcoming in this study and purified monocytes could be a better approach for such studies. However, purification of monocytes could activate these monocytes leading to changes in the expression of phenotypic markers.

MMe phenotype has been described in the adipose tissue of obese and diabetic subjects (7, 33–35). Moreover, MMe phenotype was also shown to be induced by *ex-vivo* treatment of monocytes-derived macrophages with glucose, insulin, and palmitate resulting in different macrophage phenotype than the M1 macrophages. Among these molecules, palmitate released from adipocytes was the main contributor for this metabolic activation of MMe (7). On the other hand, MMe cell surface markers expression has been found to be regulated by PPARγ and p62/SQSTM1, which are associated with alternative activation of M2 (7). Treatment of MMe with PPARγ antagonist led to an increase in pro-inflammatory cytokines secretion (IL-1β and TNFα). Thus, the anti-inflammatory effect exerted by PPARγ and p62/SQSTM1 is thought to limit MMe pro-inflammatory cytokines secretion, contributing to the chronic subclinical inflammatory state seen in the obese and T2DM (7). MMe phenotype exhibits characteristics present in M1 and M2 phenotypes and does not express the classic markers of M1 macrophages although they produce large amounts of pro-inflammatory cytokines in response to metabolic conditions. Thus, circulating PBMC in T2DM are MMe-like phenotype and these PBMC could shed the light in understanding the phenotypes and functions of metabolically activated MMe in tissues in humans.

The role of insulin as an anti-inflammatory hormone has been well-documented. Insulin exerts its anti-inflammatory effect by suppressing several pathways such as reactive oxygen species (ROS) generation, NADPH oxidase expression and intranuclear NFκB, and has a stimulatory effect on IκB expression along with a reduction in plasma concentrations of sICAM-1, MCP-1 and PAI-1 (36, 37). Obesity and T2DM are both associated with insulin resistance and hyperinsulinemia while T2DM is associated with hyperglycemia as well. CD11c was inhibited in both obese and obese T2DM, thus, suggesting insulin resistance playing a role in the regulation of this marker. On the other hand, IL-6, iNOS, TNFα, CD36, CD16, and CD169 were modulated significantly only in T2DM, suggesting that chronic hyperglycemia could be the modulator of these markers. Indeed, elevated plasma and expression of TNFα, IL-6 and CD36 levels have been observed in obese and T2DM patients, with a reported over-expression in adipose and skeletal muscle tissues and PBMC (23, 38–42). However, TNFα mRNA expression levels by PBMC were also reported to be either similar (43) or decreased in obesity (44) as well as measured by RT-qPCR. Similarly, IL-6 mRNA expression by PBMC was reported to be decreased in obesity (44). The general lack of correlation between altered serum levels and altered PBMC gene expression in obesity may suggest that PBMC may not be the source of aberrant serum cytokine levels. As mentioned before, the heterogeneity of cells utilized in this study represents a significant shortcoming in this study. For example, iNOS in human PBMC is expressed mostly by lymphocytes (45) while others showed iNOS expression in whole blood is predominant in monocytes (46). This heterogeneity could explain these controversial results in obesity.

Metformin glucose lowering effect is mainly exerted by inhibition of hepatic glucose production (gluconeogenesis), which is primarily associated with the activation of AMP-activated protein kinase (AMPK) (47) and to a lesser extent by increasing glucose uptake and utilization by skeletal muscle and adipose tissue (48). It also improves insulin sensitivity via targeting AMPK (49) and reduction of inflammation by antagonizing NFκB through inhibition of PI3K-Akt pathway (50). NFκB pathway in macrophages controls pro-inflammatory cytokine secretion such as IL-1β, IL-6, and TNFα. On the other hand, Metformin enhances the protein expression of anti-inflammatory cytokines IL-4 and IL-10 (49, 51). Results from this study demonstrated clearly that Metformin reversed MMe-like phenotype in circulation of T2DM as it significantly suppressed CD68, IL-6, iNOS, TNFα, and CD36 expression and increased CD11c, CD163 and CD169 expression in T2DM.

Several uncommitted (M0) macrophages (pan-macrophage) markers were examined and compared among the four groups studied. CD68 is one of the cell surface receptors that are exhibited on the surface of monocytes, naive macrophages, M1, M2, and MMe. CD68 which is known as scavenger receptor D has the ability to bind to oxidized low-density lipoproteins (LDL) and plays a major role in the development of atherosclerosis (52). CD14 along with CD68 have been used as pan-macrophage markers since they are expressed in both M1 and M2 phenotypes. CD14 expression in obesity and T2DM is controversial. The expression of CD14 in monocytes was reported to be higher in obese subjects when compared with lean subjects as measured by flow cytometry using anti-CD14 antibodies following PBMC isolation with ficoll-hypaque (53). On the other hand, a 3-fold reduction in the expression of CD14 in patients with T2DM was observed as measured by flow cytometry following PBMC isolation (54). As indicated before, these contradictions could stem from discrepancies in reliable gating strategies for flow cytometric characterization and antibody specificities. In our study, CD14 expression was comparable in the four groups studied as measured by RT-qPCR. The other pan-macrophage marker, CD11b, was also investigated in this study. CD11b is exclusively expressed on the surface of many myeloid-cells including, monocytes, macrophages, granulocytes, and to a lesser extent by natural killer cells (55). Recently, CD11b showed unexpected role in obesity-induced insulin resistance by limiting the proliferation and alternative activation of adipose tissue macrophages (ATMs) by inhibiting the IL-4/STAT6 signaling pathway. More importantly, ablation of CD11b has shown to decrease insulin resistance, which can be therapeutically beneficial (56). However, CD11b mRNA expression in our study was lower in circulating PBMC of T2DM. Further studies are needed to explore the role of CD11b in insulin resistance.

CD163, expressed by monocytes and macrophages, is important in resolution of inflammation. Although many consider CD163 as a pan-macrophage marker, studies have demonstrated a role for CD163 in immune regulation as it is greatly induced by anti-inflammatory agents such as steroid, while induction of immunosuppressant resulted in its downregulation (57). This anti-inflammatory characteristics of CD163 is supported by its increased expression in response to IL-4, and IL-10, while it was shown to be reduced in expression following exposure to TNFα and IFNγ (58). Several *in vitro* studies, characterized CD163 as an M2 marker (58, 59). Our results are consistent with the anti-inflammatory role of CD163 as it is reduced in T2DM. On the other hand, CD206 has been described as an M2 specific marker and our data demonstrated an increased expression levels of CD206 in PBMC of T2DM. These controversial results of CD206 could stem from the fact that PBMC is a heterogenous mix of monocytes, dendritic cells and lymphocytes which all express CD206. Thus, the results of CD206 cannot be interpreted correctly. Interestingly, increased CD206 from whole blood cells were increased in patients with active adult-onset Still's disease (AOSD), a rare systemic autoinflammatory disease when compared to healthy controls. The increased expression of CD206 was in lymphocytes of AOSD patients (60). The true expression of CD206 in T2DM remains to be elucidated.

A clear understanding of the effects of aging on macrophage polarization is relevant to many different diseases and biological diseases including insulin resistance (61). However, little is known about how age influences the ability of macrophage to change phenotypes in response to environmental factors and the data are contradictory as these studies examined M1 and M2 polarization in mice with different ages following isolation and *in vitro* stimulation. Mahbub et al. (62) polarized isolated splenocytes enriched for macrophages from young and aged female mice. They found decreased iNOS, IL-1β, and TNFα protein levels in aged M1 after stimulation with LPS or IFN-γ and TNFα, compared to young M1. On the other hand, an increased response to inflammatory stimuli in aged compared to younger rat M1, with significantly higher levels of TNFα mRNA were observed (63). Similar results were reported in isolated primary bone marrow macrophages from mice with different ages, TNF-α mRNA and protein secretion were significantly upregulated in aged M1 after INF-γ exposure. Arginase 1 and CD206 mRNA expression were still upregulated with IL4 stimulation in aged macrophages, but to a lesser extent than those from younger animals while IL-1ra secretion did not increase accordingly in aged mice (64). Toba et al showed similar levels of pro-inflammatory M1 markers (CCL5, CCL3, TNFα) in isolated peritoneal macrophages from young and old mice; however, peritoneal macrophages displayed reduced anti-inflammatory M2 markers (Arginase 1 and MRC1 mannose receptor C-type 1) (65). The age in the four groups in our study were significantly higher in T2DM. However, there were no correlation between age and any of the markers examined in our study suggesting that age does not play a role in the expression levels of these markers.

In conclusion, the metabolically activated circulating PBMC in T2DM are MMe-like cells which have distinct and different phenotype characteristics than the one present in the lean or obese subjects. MMe-like circulating PBMC express increased levels of CD68, CD16, IL-6, iNOS, TNFα, CD206 and CD36 and decreased levels of CD11b, CD11C, CD169, and CD163. Metformin treatment in T2DM inhibits MMe-like phenotype and may exert its known anti-atherosclerotic effect through this mechanism. Further studies are required to elucidate the expression levels of other markers in obesity and T2DM and examine the characteristics of these MMe-like phenotypes resulting from insulin resistance and hyperglycemia separately. Future studies utilizing isolated circulating monocytes from T2DM are needed to characterize MMe like monocytes which data suggest to belong to the “intermediate monocytes” phenotype as they express high levels of CD14 and CD16.

## Author contributions

AhA idea conceived by him; AhA and MA analyzed data and wrote and drafted the manuscript; HA, RanA, NA, and RahA did literature review, sample analysis, and data collection; AwA, MZ, AN, and AA-B contributed to the data analysis and drafting and reviewing of the final manuscript; All authors approved the final format of the submitted manuscript.

### Conflict of interest statement

The authors declare that the research was conducted in the absence of any commercial or financial relationships that could be construed as a potential conflict of interest.
